# 671. MORE FUN: Progressive Mobility, Endurance, Strength, and Function for Burned Children

**DOI:** 10.1093/jbcr/irag033.552

**Published:** 2026-04-06

**Authors:** Ndidi C Okeke, Ingrid Parry, Jason Heard, Kathleen S Romanowski, Tina L Palmieri, Soman Sen

**Affiliations:** Shriners Children's - Northern California, Sacramento, CA; Shriners Children's - Northern California, Sacramento, CA; Shriners Children's - Northern California, Sacramento, CA; Shriners Children's - Northern California, Sacramento, CA; University of California Davis Health, Shriners Children’s - Northern California, Sacramento, CA; Shriners Children's - Northern California, Sacramento, CA

## Abstract

**Introduction:**

Physical rehabilitation is a mainstay in the treatment of acutely injured burn patients. Despite the long-standing practice of implementing physical and occupational therapy in burn injury treatment, there are no standard therapy practices, prescribed daily duration, or timing that are established. The main aim of this investigation was to determine if a prescribed early physical rehabilitation program with less bedrest improves physical rehabilitation for acutely injured pediatric burn patients.

**Methods:**

We performed a single center randomized control prospective study of pediatric patients (3-18 years of age) with an acute burn injury. To be included in the study patients needed to have a survivable burn injury of 5% of total body surface area (TBSA) or more and have an anticipated need for skin grafting. Patients were randomized to receive either active therapy (AT) or standard therapy (ST). The AT group received directed therapy of 45 to 60 minutes per day, with no pauses in the perioperative or postoperative period. ST group received usual therapy and care guided by the treating surgeon. Primary outcome was the 6-minute walk test (6MWT) distance at discharge. Secondary outcomes included Time-Up-and-Go, Patient Reported Outcome Measurement Information System scores, Canadian Occupational Performance Measure, and post discharge activity.

**Results:**

52 patients were enrolled in the study: 28 patients were randomized to the ST group and 24 patients were randomized to the AT group. Mean age (7.3 ± 4.7 vs. 8.3 ± 4.7 years), total burn TBSA (27 ± 21 vs. 21 ± 15%), and third degree burn TBSA (19 ± 16 vs. 16 ± 17%) did not differ between the groups (ST vs AT). While not reaching statistical significance (*p*=.141), the AT group walked longer distances (+41 meters) during the 6MWT at discharge and demonstrated more time in a “very active” activity zone during the 3 months after discharge (AT: 30%, ST; 20%, *p*<.001). There were no differences in skin graft loss or complications between the groups.

**Conclusions:**

Intensive, prescribed, and daily physical therapy intervention can be safely administered for acutely burn-injured pediatric patients without complications. These interventions may help improve activity after discharge.

**Applicability of Research to Practice:**

Pediatric burn patients can and should participate in aggressive daily physical therapy. Historically, concerns for graft loss and other perioperative wound healing complications have tended towards bedrest and limited mobility in this vulnerable population. However, this study demonstrates scheduled active therapy is not only safe but may benefit patients as well.

**Funding for the study:**

Institutional research grant.

